# Integrating *E*-nose, *E*-tongue, and HS-SPME-GC–MS analysis reveals the volatile profile and flavor characteristics of different Wampee (*Clausena lansium* (Lour.) Skeels)

**DOI:** 10.1016/j.fochx.2026.104660

**Published:** 2026-10-28

**Authors:** Qingshan Li, Chunyan Zhang, Ruibing Xu, Gengrui Zhu, Yi Chen, Haidong Huang, Gaoyang Zhang

**Affiliations:** a Guangxi Key Laboratory of Agro-environment and Agro-products Safety, National Demonstration Center for Experimental Plant Science Education, College of Agriculture, Guangxi University, Nanning 530004, Guangxi, China; b School of Management, SEGi University, Petaling Jaya 47810, Selangor, Malaysia; c Key Laboratory Forest Tree Genetics & Breeding of Liaoning Province, College of Forestry, Shenyang Agricultural University, Shenyang 110866, China; d College of Life Sciences, Shangrao Normal University, Shangrao 334001, China; e Institute of Precision Medicine, Zhejiang Chinese Medical University, Hangzhou 311400, China

**Keywords:** Wampee (*Clausena lansium*), Volatile metabolites, Electronic nose and tongue, Odor activity value (OAV), Terpenoid biosynthesis

## Abstract

Wampee (*Clausena lansium*), a nutrient-rich tropical fruit in the Rutaceae family, is prized across southern China and Southeast Asia for its distinctive sweet-tart flavor and aromatic complexity, yet the chemical basis of its varietal divergence remains poorly characterized. Here, we present a multi-platform integrated flavor-omics strategy that combines electronic nose profiling, electronic tongue sensing, and headspace solid-phase microextraction gas chromatography–mass spectrometry (HS-SPME-GC–MS) to systematically resolve the volatile and taste architectures of three representative wampee cultivars—’Shanhuangpi’, ‘Bingtangxin’, and ‘Heijingang’—across peel and flesh tissues. Electronic nose analysis revealed that the first two principal components explained 90.98% of the total variance among the six sample groups, with linear discriminant analysis achieving 100% classification accuracy driven predominantly by long-chain alkane, sulfur-containing, and methylated volatile classes. Electronic tongue profiling identified sourness, sweetness, and aftertaste-astringency as the primary discriminants, and uncovered a consistent tissue-partitioning pattern whereby flesh tissues are enriched in sourness and sweetness, whereas peel accumulates astringency and bitterness. HS-SPME-GC–MS identified 288 volatile compounds spanning 15 chemical classes, with terpenoids constituting 28.82% of the total volatile pool. Odor activity value (OAV) analysis of 57 quantitated volatiles identified five key odorants—myristicin (4-Methoxy-6-(2-Propenyl)-1,3-Benzodioxole), estragole, damascenone, linalool, and myrcene—that collectively define the characteristic balsamic, floral, spicy, woody, and fruity sensory notes of wampee. Partial least squares-discriminant analysis robustly segregated peel from flesh (R^2^Y = 0.833, Q^2^ = 0.647), with 96 volatile metabolites significantly up-regulated in peel and enriched in monoterpene hydrocarbons. KEGG pathway enrichment—an indirect inference from volatile end-products—indicated that terpenoid backbone, monoterpenoid, and sesquiterpenoid/triterpenoid biosynthesis are the principal routes plausibly governing tissue-specific aroma formation. These findings establish a foundation for wampee quality assessment, cultivar authentication, and flavor-oriented breeding.

## Highlights

• Integrated *E*-nose, E-tongue, and HS-SPME-GC–MS analysis revealed volatile profiles of Wampee.
• Terpenoids constitute 28.82% of 288 identified volatile compounds, and OAV analysis identifies five key odorants—myristicin, estragole, damascenone, linalool, and myrcene.
• KEGG pathway enrichment and PLS-DA modeling reveal that tissue-specific aroma in wampee is governed by differential engagement of terpenoid backbone, monoterpenoid, and sesquiterpenoid biosynthesis pathways.

## 1 Introduction

Wampee (*Clausena lansium* (Lour.) Skeels), a member of the Rutaceae family, is an underutilized tropical and subtropical fruit native to southern China and Southeast Asia (Ding et al., 2025). The fruit is characterized by its distinctive aromatic profile, attractive yellowish-brown to dark purple-black peel, and translucent, juicy flesh with a unique sweet-tart flavor (Chokeprasert et al., 2007). Despite its growing economic importance, wampee remains less studied compared to other tropical fruits such as mango and lychee, particularly regarding the systematic characterization of its volatile compounds and the underlying metabolic pathways that govern flavor formation (Ding et al., 2025).

The fruit is recognized for its diverse nutritional and bioactive constituents, including high levels of vitamin C, flavonoids, polyphenols, and essential oils (Zeng et al., 2025). These bioactive compounds have been associated with various health-promoting effects, such as antioxidant, anti-inflammatory, and antimicrobial properties, making wampee a promising functional food (Ding et al., 2025; Zeng et al., 2025). However, the commercial value and consumer acceptance of wampee are predominantly determined by its flavor quality, which is fundamentally governed by the complex composition of volatile organic compounds (VOCs) and non-volatile taste-active metabolites (Dadwal et al., 2024). Flavor is a multidimensional perceptual experience that integrates aroma and taste sensations (Wang et al., 2023). The aroma of fruit is primarily contributed by VOCs, including terpenes, esters, aldehydes, alcohols, and ketones (Wang et al., 2022). These compounds are biosynthetically derived from three major metabolic pathways: the terpenoid biosynthesis pathway, phenylalanine metabolism, and fatty acid biosynthesis pathway (Dadwal et al., 2024). The relative abundance and composition of VOCs are dynamically regulated by fruit genotype, developmental stage, and postharvest handling conditions, resulting in the characteristic aroma profiles that define consumer preference (Dadwal et al., 2024). The molecular regulatory mechanisms that link volatile biosynthesis to the accumulated aroma phenotype—including the coordinate action of structural genes and transcription factors—are now being resolved through combined transcriptomic and metabolomic approaches (Huang et al., 2025; Liang et al., 2024).

In wampee specifically, preliminary studies have identified a range of volatile compounds that contribute to its distinctive aroma. Chokeprasert et al. (2007) analyzed the volatile components of wampee leaves, pericarp, and seeds, identifying 39 components in the leaf, 27 in the pericarp, and 25 in the seeds, with sabinene being the predominant compound across different plant parts. This study demonstrated that the volatile composition varies significantly among different tissues, highlighting the importance of tissue-specific analysis for comprehensive flavor characterization (Chokeprasert et al., 2007). Ma et al. (2021) further investigated the chemical composition of essential oils from wampee seeds, revealing significant antifungal activity against Candida strains, which was correlated with the major volatile constituents. These studies indicate that wampee tissues contain distinct volatile profiles that may contribute to both flavor quality and bioactive properties. More recent work on whole wampee fruit has shown that the aroma and taste properties differ markedly among fruit parts (Zhao et al., 2023), and that the phenolic metabolome of wampee can be resolved by targeted metabolomics combined with OPLS-DA (Tu et al., 2026.

Traditional analytical methods for characterizing fruit volatile compounds have relied heavily on gas chromatography–mass spectrometry (GC–MS) coupled with various extraction and preconcentration techniques (Pang et al., 2023). Among these, headspace solid-phase microextraction (HS-SPME) has emerged as a preferred sample preparation method due to its solvent-free nature, simplicity, and ability to concentrate trace-level volatile compounds from complex food matrices (Lei et al., 2026). However, these conventional analytical techniques, while providing detailed chemical composition data, are often time-consuming, labor-intensive, and unsuitable for rapid, high-throughput quality assessment (Vanaraj et al., 2025). Moreover, GC–MS analysis typically focuses on volatile compounds and provides limited information about the taste-active non-volatile metabolites that equally contribute to overall flavor perception (Lu et al., 2022).

In recent years, electronic nose (*E*-nose) and electronic tongue (E-tongue) technologies have emerged as promising rapid, non-destructive alternatives for food quality evaluation (Vanaraj et al., 2025). An *E*-nose simulates the human olfactory system by employing an array of gas sensors to detect and differentiate complex volatile profiles, while an *E*-tongue mimics the human gustatory system using electrochemical sensor arrays to characterize taste-active compounds (Lu et al., 2022). Both technologies generate fingerprint-like response patterns that, when combined with appropriate chemometric methods, can effectively classify samples, monitor freshness, and predict quality attributes (Vanaraj et al., 2025). Numerous studies have demonstrated the successful application of *E*-nose and E-tongue systems for quality assessment of various food products (Lu et al., 2022; Vanaraj et al., 2025). For fruit quality evaluation specifically, E-nose systems have been used to monitor ripening stages, detect fungal infections, and differentiate cultivars based on aroma profiles (Wang et al., 2022). Similarly, E-tongue systems have been applied to assess sweetness, sourness, bitterness, and umami taste attributes in fruits (Vanaraj et al., 2025).

Despite these advances, single-sensor systems have inherent limitations that can compromise their accuracy and reliability in complex food matrices. *E*-nose sensors are susceptible to drift and humidity interference, while E-tongue sensors may struggle to differentiate similar taste-active compounds (Calvini & Pigani, 2022). Furthermore, neither technology alone can provide the detailed molecular identification and quantification of specific compounds that traditional chromatographic methods offer (Vanaraj et al., 2025). To overcome these limitations, recent research has increasingly focused on integrating multiple analytical techniques and data fusion strategies that combine the complementary strengths of different sensing modalities. Data fusion of *E*-nose and E-tongue data has been shown to improve classification accuracy by enabling more robust discrimination of food samples with subtle quality differences (Hou et al., 2023). Additionally, combining sensor-based methods with detailed chromatographic profiling offers a powerful approach to correlate rapid sensor responses with specific chemical markers, facilitating the interpretation of sensor data in terms of underlying biochemical changes (Cannavacciuolo et al., 2023).

For wampee specifically, research on flavor chemistry and metabolite profiling remains limited (Ding et al., 2025). While Chokeprasert et al. (2007) and Ma et al. (2021) profiled volatile components in wampee tissues using GC–MS, the integrated application of *E*-nose, E-tongue, and HS-SPME-GC–MS for comprehensive characterization of wampee peel and flesh volatiles has not been reported. Furthermore, the comparison of volatile profiles between peel and flesh tissues, and the elucidation of tissue-specific metabolic pathways that govern volatile biosynthesis in wampee, remain unexplored (Ding et al., 2025). Given that peel and flesh tissues have distinct physiological functions and biochemical compositions, they may also differ significantly in their volatile profiles and metabolic pathways (Wang et al., 2022). Understanding these differences is essential not only for optimizing postharvest handling and processing practices but also for enabling targeted breeding and quality improvement strategies that enhance the flavor quality of wampee fruit.

In this context, the present study aims to address these gaps by implementing an integrated analytical approach that combines E-nose, E-tongue, and HS-SPME-GC–MS to investigate the volatile profiles of three characteristic wampee cultivars from Guangxi Zhuang Autonomous Region, China. Our objectives are to: (1) establish volatile fingerprints for each cultivar, (2) identify key aroma-active compounds in peel and flesh and their sensory contributions, and (3) elucidate the chemical basis of the observed aroma differences. These findings will provide a scientific foundation for quality assessment, cultivar identification, and value-added processing of wampee.

## 2 Materials and methods

### 2.1 Plant materials

Three wampee (*Clausena lansium* (Lour.) Skeels) cultivars—’Shanhuangpi’, ‘Bingtangxin’, and ‘Heijingang’—were obtained from the Guangxi Academy of Agricultural Sciences, Nanning, Guangxi Zhuang Autonomous Region, China (22°49′27.8″N, 108°17′58.2″E). Three ten-year-old trees were selected for each cultivar. On each tree, four reference fruits with uniform growth characteristics were tagged at the east, west, south, and north orientations. Based on this reference framework, ten fruits were harvested from each tree, and fruits collected from each single tree were pooled to form one biological replicate, generating three pooled biological replicates for each cultivar. After harvesting, peel and flesh tissues were separately collected for each pooled sample. Fresh peel and flesh samples were immediately used for electronic nose (*E*-nose), electronic tongue (E-tongue), and soluble solids content (SSC) measurements. The remaining peel and flesh tissues were flash-frozen in liquid nitrogen and stored at −80 °C for subsequent metabolomic analysis. The data have been subjected to mean-centering prior to statistical analysis. Sample abbreviations are listed in Table 1.

**Table 1:** Wampee (*Clausena lansium* (Lour.) Skeels) sample information.

Sample code	Cultivar	Tissue	Species
SF	Shanhuangpi	Flesh	*Clausena lansium* (Lour.) Skeels
SP		Peel	
ZF	Heijingang	Flesh	
ZP		Peel	
TF	Bingtangxin	Flesh	
TP		Peel	

Three cultivars (‘Shanhuangpi’, ‘Bingtangxin’ and ‘Heijingang’) were each sampled as peel and flesh, giving six sample groups. Each sample group comprised three pooled biological replicates. Samples are coded by cultivar (S, ‘Shanhuangpi’; T, ‘Bingtangxin’; Z, ‘Heijingang’) followed by tissue (P, peel; F, flesh).

**Fig. 1:**
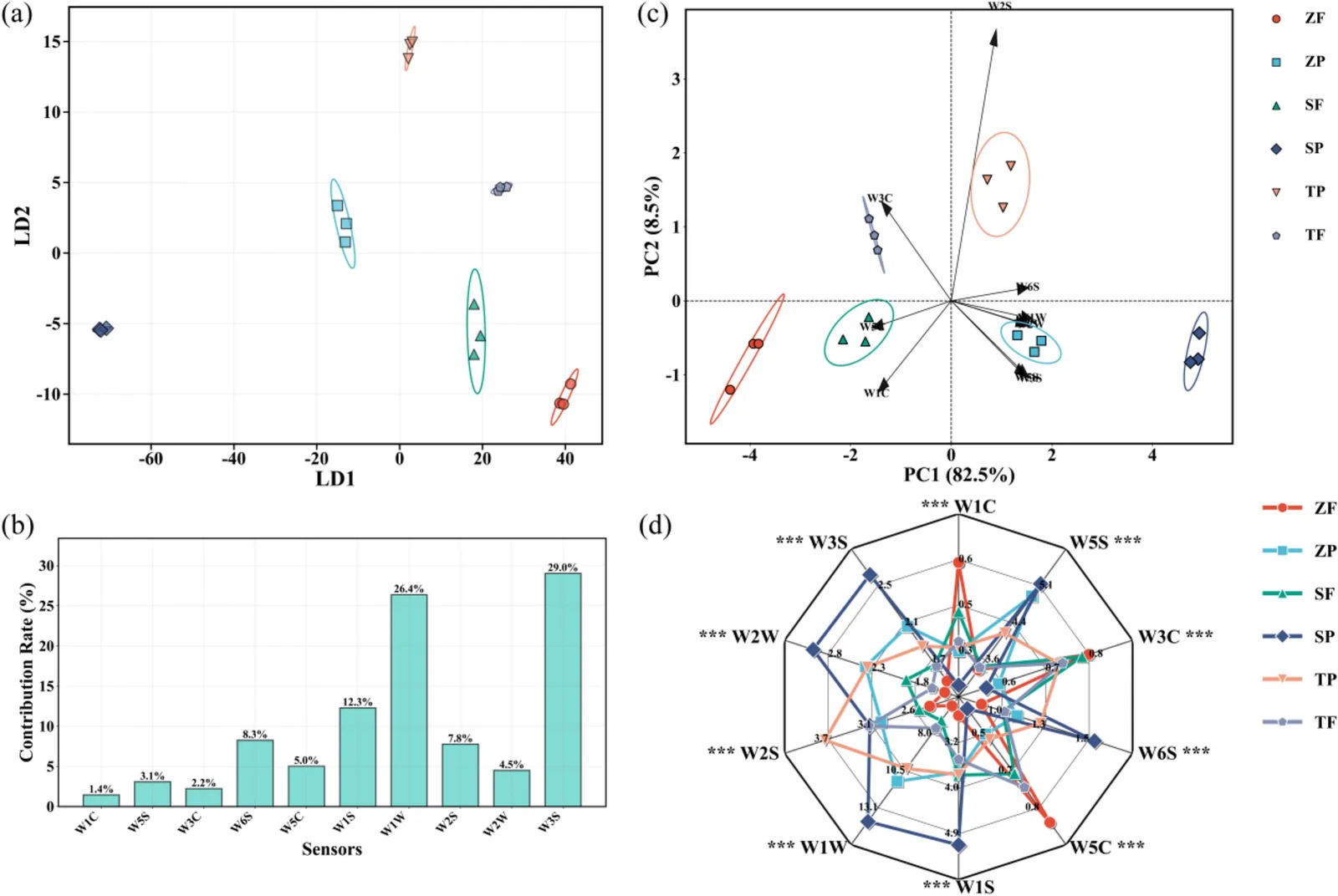
Electronic nose (PEN3) characterization of peel and flesh tissues of three wampee (*Clausena lansium* (Lour.) Skeels) cultivars. (a) Linear discriminant analysis (LDA) of the ten-sensor response matrix for the six sample groups (SP, SF, TP, TF, ZP, ZF); (b) relative contribution (%) of each linear discriminant to the total discrimination; (c) principal component analysis (PCA) score plot of the *Z*-score-standardized sensor responses (PC1 = 82.50%, PC2 = 8.49%); (d) radar plot of the mean normalized response of each of the ten metal-oxide sensors (W1C, W5S, W3C, W6S, W5C, W1S, W1W, W2S, W2W, W3S) in each sample group. Sample abbreviations: S, ‘Shanhuangpi’; T, ‘Bingtangxin’; Z, ‘Heijingang’; P, peel; F, flesh.

### 2.2 Determination of quality parameters

Soluble Solids Content (SSC) was measured using a digital refractometer (PAL-1, Atago, Japan) at 20 °C. The juice was directly applied to the prism, and the reading was expressed as a percentage (%). Each sample was measured three times.

Titratable Acidity (TA) was determined by the NaOH titration method. Briefly, 10.0 mL of juice was diluted with 50 mL of distilled water and titrated with 0.1 mol/L NaOH standard solution using phenolphthalein as an indicator. The endpoint was determined when the solution turned light pink and remained stable for 30 s. TA was calculated as malic acid equivalents and expressed as a percentage. Each sample was analyzed in triplicate.

Calculation formula:

$$\mathrm{TA}\;\left(\%\right)=\frac{\left(V\times C\times 67.04\right)}{V_{\text{sample}}\times 10}$$

where: V = volume of NaOH consumed (mL); C = concentration of NaOH (mol/L); 67.04 = molar mass of malic acid (g/mol); $V_{\mathrm{sample}}$ = sample volume (mL).

Soluble sugar content was determined using the dinitrosalicylic acid (DNS) method with minor modifications. Briefly, 0.5 mL of juice was mixed with 1.5 mL of DNS reagent and heated in a boiling water bath for 5 min. After cooling to room temperature, the absorbance was measured at 540 nm using a UV–Vis spectrophotometer (UV-1800, Shimadzu, Japan). A standard curve was prepared using sucrose solutions of known concentrations. Soluble sugar content was expressed as a percentage. Each sample was analyzed in triplicate. The sugar-acid ratio was calculated as the ratio of soluble sugar content to titratable acidity.

### 2.3 Electronic nose measurement

Odor profiling was performed using a PEN3 portable electronic nose system (AirSense Analytics GmbH, Germany) equipped with a 10 metal-oxide sensor array, each exhibiting selective responsiveness to distinct classes of volatile compounds. The sensor array comprises W1C (aromatic compounds), W5S (nitrogen oxides; featuring a broad-spectrum response), W3C (ammonia and aromatic compounds), W6S (hydrogen), W5C (alkanes and aromatic compounds), W1S (methane; featuring a broad-spectrum response), W1W (sulfides and organic compounds), W2S (ethanol and broad-spectrum alcohols), W2W (aromatic compounds and organosulfur compounds), and W3S (alkanes and methane). Briefly, 1.0 g sample was placed in a dedicated headspace vial, equilibrated at 40 °C for 30 min, and analyzed with the following parameters: cleaning time 120 s, sample preparation time 5 s, injection flow rate 400 mL/min, measurement time 120 s, cleaning flow rate 400 mL/min. Sensor responses were recorded as G/G₀ (G: sample response; G₀: baseline in clean air). For sensors W1C, W3C, and W5C, response values were inverted to maintain consistency. The PEN3 metal-oxide semiconductor sensors are broadly cross-reactive; the nominal chemical-class labels assigned to each channel (e.g., sulfur compounds, methyl compounds) describe predominant rather than exclusive sensitivities and are used here only as a convenient shorthand for the composite response each sensor delivers. Each sample was measured in triplicate. Data were analyzed by one-way ANOVA with Tukey's HSD test (*p* < 0.05). Principal component analysis (PCA) and linear discriminant analysis (LDA) were performed on *Z*-score standardized data using Python 3.8.

### 2.4 Electronic tongue measurement

Taste profiling was performed using an SA402B electronic tongue system (INSENT, Japan) equipped with a nine-sensor array responsive to distinct taste attributes. The sensor array comprised AAE (sourness), CT0 (bitterness), CA0 (astringency), GL1 (aftertaste bitterness), C00 (aftertaste astringency), AE1 (umami), BT0 (richness), HA0 (saltiness), and SW0 (sweetness). Sample preparation involved homogenizing 1.0 g of sample with deionized water, followed by centrifugation at 4000 ×*g* for 10 min and filtration through a 0.45 μm membrane. Sensors were conditioned prior to measurement by immersion in conditioning solution for 30 s per cycle over 20 cycles, with a stability threshold of 0.5 mV. Each sample was measured in triplicate, with each measurement lasting 30 s. Sensor responses were recorded as potential differences (mV) relative to the reference solution. Data were analyzed by one-way ANOVA with Tukey's HSD test (*p* < 0.05). Principal component analysis (PCA) and linear discriminant analysis (LDA) were performed on Z-score standardized data using Python 3.8 (Table 2).

**Table 2:** Sensor configuration and taste response characteristics of the INSENT SA402B electronic tongue system used for taste profiling of wampee peel and flesh.

Sensor code	Taste attribute	Description	Response characteristics
AAE	Sourness	Acidic taste	Responds to acid and sour taste compounds
CT0	Bitterness	Bitter taste	Responds to bitter compounds (caffeine, quinine)
CA0	Astringency	Astringent taste	Responds to tannins and astringent compounds
GL1	Aftertaste-B	After-bitter taste	Detects lingering bitter aftertaste
C00	Aftertaste-A	After-astringent taste	Detects lingering astringent aftertaste
AE1	Umami	Savory taste	Responds to glutamate and nucleotides
BT0	Richness	Body/mouthfulness	Responds to kokumi-enhancing compounds
HA0	Saltiness	Salty taste	Responds to salt and sodium-containing compounds
SW0	Sweetness	Sweet taste	Responds to sugars and sweetening agents

### 2.5 Determination of volatile compounds by HS-SPME-GC–MS

Wampee flesh and peel tissues were ground in liquid nitrogen. Approximately 3 g of ground sample was weighed and transferred to a 20 mL headspace vial. Internal standard (2.0 μL of n-pentadecane-d32 at 250 μg/mL) and 5 mL of saturated NaCl solution were added to the vial, which was immediately sealed. Volatile compounds were extracted using headspace solid-phase microextraction (HS-SPME) with an SPME Arrow fiber (DVB/Carbon WR/PDMS, 120 μm × 20 mm, 1.1 mm diameter; Thermo Fisher Scientific, Waltham, MA, USA). The HS-SPME extraction was performed using a TriPlus RSH SMART headspace autosampler. Extraction conditions were as follows: incubation and extraction temperature 80 °C, incubation time 20 min, extraction time 10 min. The fiber was aged at 240 °C for 10 min before each extraction. Fiber desorption aging was performed for 2 min before and after each sample analysis. Sample desorption time was 1 min. The DVB/Carbon WR/PDMS (120 μm) Arrow fiber was selected because its mixed polar/non-polar phases provide a broad extraction window spanning both highly volatile esters/aldehydes and semi-volatile terpenes and phenylpropenes. The extraction temperature of 80 °C was chosen to maximize recovery of semi-volatile terpenoids while keeping the headspace dwell time short (10 min); nonetheless, such temperatures can promote thermal degradation of labile constituents (e.g., cis-3-hexenal, certain esters) or artefact formation, and any such bias could not be fully quantified in the present study.

Chromatographic separation and mass spectrometric detection were performed using a TRACE 1610 GC–Orbitrap Exploris high-resolution gas chromatography–mass spectrometry system (Thermo Fisher Scientific). Samples were injected in split mode with a split ratio of 10:1. Separation was achieved on a VF-WAXms capillary column (25 m × 0.25 mm × 0.2 μm; Agilent CP9204, Santa Clara, CA, USA). Injector temperature was set at 240 °C. High-purity helium was used as the carrier gas at a flow rate of 1.0 mL/min. Septum purge flow was 3 mL/min. The oven temperature program was as follows: initial temperature 40 °C (hold 0 min), ramped at 8 °C/min to 120 °C, then ramped at 20 °C/min to 230 °C and held for 4.5 min. Total run time was 20 min. Mass spectrometry was performed using an electron ionization (EI) ion source at 280 °C with an electron energy of 70 eV. Full scan mode was used with a mass scan range of *m*/*z* 35–500. Mass resolution was set at 30,000.

### 2.6 Qualitative and quantitative analysis of volatile organic compounds

Raw GC–MS data were processed using Compound Discovery 3.3 SP3 software. Volatile compounds were identified by matching experimental mass spectra and retention indices (RIs) against three databases: NIST 2023, Thermo Scientific GC–Orbitrap Flavor and Fragrances v1.0, and a self-built database from Majorbio. N-alkane standards (C10–C29) were analyzed under identical chromatographic conditions to calculate experimental RIs. Compound identifications were evaluated using the High-Resolution Filtering (HRF) scoring function in Compound Discovery; the screening criteria were: HRF score > 80, spectral match score > 600, and |ΔRI| < 50 between experimental and database RI values. The GC–MS chromatogram is shown in Fig. S1. Quantification was semi-quantitative, using n-pentadecane-d_32_ (250 μg/mL, 2.0 μL) as the internal standard and assuming relative response factors of unity. The remaining analytes were quantified according to the following equation:

$$X_{i}=\frac{V_{\text{is}}\times C_{\text{is}}}{V}\times \frac{A_{i}}{A_{\text{is}}}$$

where $X_{i}$ is the relative content of compound i in the liquid sample (μg/mL); $V_{\mathrm{is}}$ is the volume of internal standard added (μL); $C_{\mathrm{is}}$ is the concentration of the added internal standard (μg/mL); *V* is the volume of the liquid sample (μL); $A_{\mathrm{is}}$ is the peak area of the internal standard; and *Aᵢ* is the peak area of compound *i*.

Principal component analysis (PCA) and orthogonal partial least-squares discriminant analysis (OPLS-DA) were performed on the preprocessed data matrix using the ropls package (v1.6.2) in R. Model stability was evaluated by 7-fold cross-validation. Differentially abundant metabolites were identified based on variable importance in projection (VIP) scores from the OPLS-DA model and Student's *t*-test *p*-values, with VIP > 1 and *p* < 0.05 as the significance thresholds.

Metabolic pathway annotation of differential metabolites was performed using the Kyoto Encyclopedia of Genes and Genomes (KEGG) database (https://www.kegg.jp/kegg/pathway.html). Pathway enrichment analysis was conducted using the scipy.stats package in Python, and Fisher's exact test was applied to identify the biological pathways most relevant to the experimental treatment.

Volatile compound classification, sensory flavor description analysis, OAV, and aroma contribution index (ACI) analyses were all performed on the Majorbio biological cloud platform. All volatile compounds' sensory threshold values are based on Compilations of odor threshold values in air, water, and other media (2nd ed.) (Van Gemert, 2018). ACI represents the percentage of the threshold value for each compound variety relative to the sum of the overall compound thresholds for that variety. The OAV of each volatile compound was calculated as the ratio of its measured concentration (or relative peak area) to its odor detection threshold in the corresponding matrix, according to the equation

$${\mathrm{OAV}}_{i}=\frac{C_{i}}{{\mathrm{OT}}_{i}}$$

$C_{i}{\mathrm{OT}}_{i}$ where is the concentration of compound i and is the odor threshold of compound *i*. Compounds with OAV > 1 were considered to make a direct contribution to the overall aroma profile, whereas compounds with OAV < 1, although detectable by GC–MS, were deemed to have no practical sensory impact. This study selected compounds with OAV ≥ 1 for subsequent analysis.

To quantify the relative contribution of each odor-active compound to the overall aroma profile, air was selected as the medium; the unit of concentration was μg/mL, and the ACI was calculated as

$${\mathrm{ACI}}_{i}=\frac{{\mathrm{OAV}}_{i}}{\sum_{j=1}^{n}\mathrm{OA}V_{j}}\times 100\%$$

where $\sum_{j=1}^{n}\mathrm{OA}V_{j}$ is the sum of OAVs across all detected volatile compounds (n). Compounds with higher ACI values were identified as the key odorants driving cultivar-specific or tissue-specific aroma characteristics. *ACI* was calculated separately for each sample.

### 2.7 Data analysis

All data were expressed as mean ± standard deviation (SD) of three independent replicates. Significant differences among cultivars were analyzed by one-way analysis of variance (ANOVA) followed by Duncan's multiple range test using SPSS software (version 26.0, IBM, USA). Differences were considered statistically significant at *P* < 0.05. Correlation analysis was performed using Pearson's correlation coefficient. PCA was conducted to evaluate the overall quality differences among cultivars.

## 3 Results and discussion

### 3.1 Electronic nose sensor response and multivariate analysis

Volatile profiles of six samples were characterized using a PEN3 electronic nose equipped with 10 metal-oxide semiconductor sensors (W1C, W5S, W3C, W6S, W5C, W1S, W1W, W2S, W2W, W3S). All samples exhibited the strongest responses on W1W (sulfur compounds), W5S (nitrogen oxides), and W1S (methyl compounds), with SP showing the highest W1W response (14.01 ± 0.29) and ZF the lowest (6.07 ± 0.02). Principal component analysis (PCA) of the sensor response data revealed that the first two principal components (PC1 and PC2) collectively explained 90.98% of the total variance (PC1: 82.50%; PC2: 8.49%). All six samples were well separated in the PC1–PC2 score plot, with 95% confidence ellipses showing no overlap between groups. The loading plot indicated that W1W, W2W, and W3S contributed most to PC1, whereas W2S, W3C, and W1C dominated PC2. Linear discriminant analysis (LDA) achieved an overall classification accuracy of 100%, with no misclassification observed. Feature importance analysis identified W3S (29.0%), W1W (26.4%), and W1S (12.3%) as the top contributing sensors, indicating that the composite responses of W3S, W1W, and W1S were the principal drivers of sample discrimination.

The pronounced responses of W1W, W5S, and W1S across all six samples indicate broad differences in the overall volatile profile among the six sample groups, reflecting composite sensor responses rather than specific chemical classes; chemical-class interpretation is therefore deferred to the GC–MS correlation data, where W1W—nominally a sulfur sensor—correlated most strongly with phenolics rather than with sulfur-containing compounds. This sensor response pattern is consistent with previous PEN3 *E*-nose applications to fruit aroma profiling, where W1W and W1S consistently emerge as the most discriminating sensors due to the prevalence of sulfur-containing esters and methyl-containing terpenoids in fleshy fruit volatile blends (Du et al., 2019; Feng et al., 2025). The exceptionally high variance explained by PCA (90.98%) reflects the substantial inter-sample divergence in overall volatile emission profiles, which in turn enables the perfect LDA classification accuracy—a result that parallels the 100% classification rates reported for PEN3-based discrimination of plant-based drinks and fruit cultivars using LDA on sensor array responses (Ali et al., 2023; Papp et al., 2024). The feature importance analysis further reveals that W3S, W1W, and W1S together account for 67.7% of the total discriminant power, suggesting that these sensor channels—rather than the full volatile complement—are the primary drivers of sample-to-sample sensory distinction. This finding aligns with integrated *E*-nose–GC–MS studies that identify a comparatively small subset of sensor channels as robust predictors of fruit volatile phenotype, thereby offering a parsimonious basis for rapid quality screening (Zhang et al., 2024). The per-cultivar mean E-nose sensor responses for the six wampee sample groups are summarized in Table S1.

### 3.2 Electronic tongue sensor response and multivariate analysis

An INSENT SA402B *E*-tongue system was used to characterize the taste profiles of six samples. Linear discriminant analysis (LDA) achieved complete separation among all six groups (Fig. 2a), indicating that each cultivar combination possesses a distinctive sensory fingerprint that can be unambiguously discriminated. Across all varieties, sourness (23.5%), sweetness (18.4%), and aftertaste-astringency (15.3%) were the dominant attributes and the principal contributors to sample discrimination, whereas the remaining six tastes–astringency, bitterness, umami, saltiness, richness, and aftertaste-bitterness–each contributed 2.7–14.2% (Fig. 2b). The per-cultivar mean E-tongue taste responses for the six wampee sample groups are summarized in Supplementary Table S2. Principal component analysis (PCA) of the E-tongue data further separated the samples, with the first two components explaining 91.76% of the total variance (PC1, 81.85%; PC2, 9.91%) (Fig. 2c). Bitterness, umami, and sweetness loaded most strongly on PC1, while saltiness, sourness, and aftertaste-bitterness dominated PC2. Taste intensities differed markedly between wampee peel and flesh (Table S3): TF showed the highest umami (9.65 ± 0.35) and sweetness (6.14 ± 0.43) responses, with umami, saltiness, and richness as its core attributes; SP was dominated by astringency (5.15 ± 0.37) and bitterness, with astringency, saltiness, and richness as its core attributes; TP exhibited the strongest saltiness (8.08 ± 0.46); and ZF, SF, and ZP were characterized primarily by umami, by umami plus richness, and by saltiness plus richness, respectively (Fig. 2d). Notably, the flesh samples generally exhibited higher intensity in sourness and sweetness compared to their corresponding peel counterparts, whereas peel tissues showed elevated astringency and bitterness, suggesting that tissue-dependent metabolic partitioning of flavor-active compounds underlies the observed sensory divergence.

**Fig. 2:**
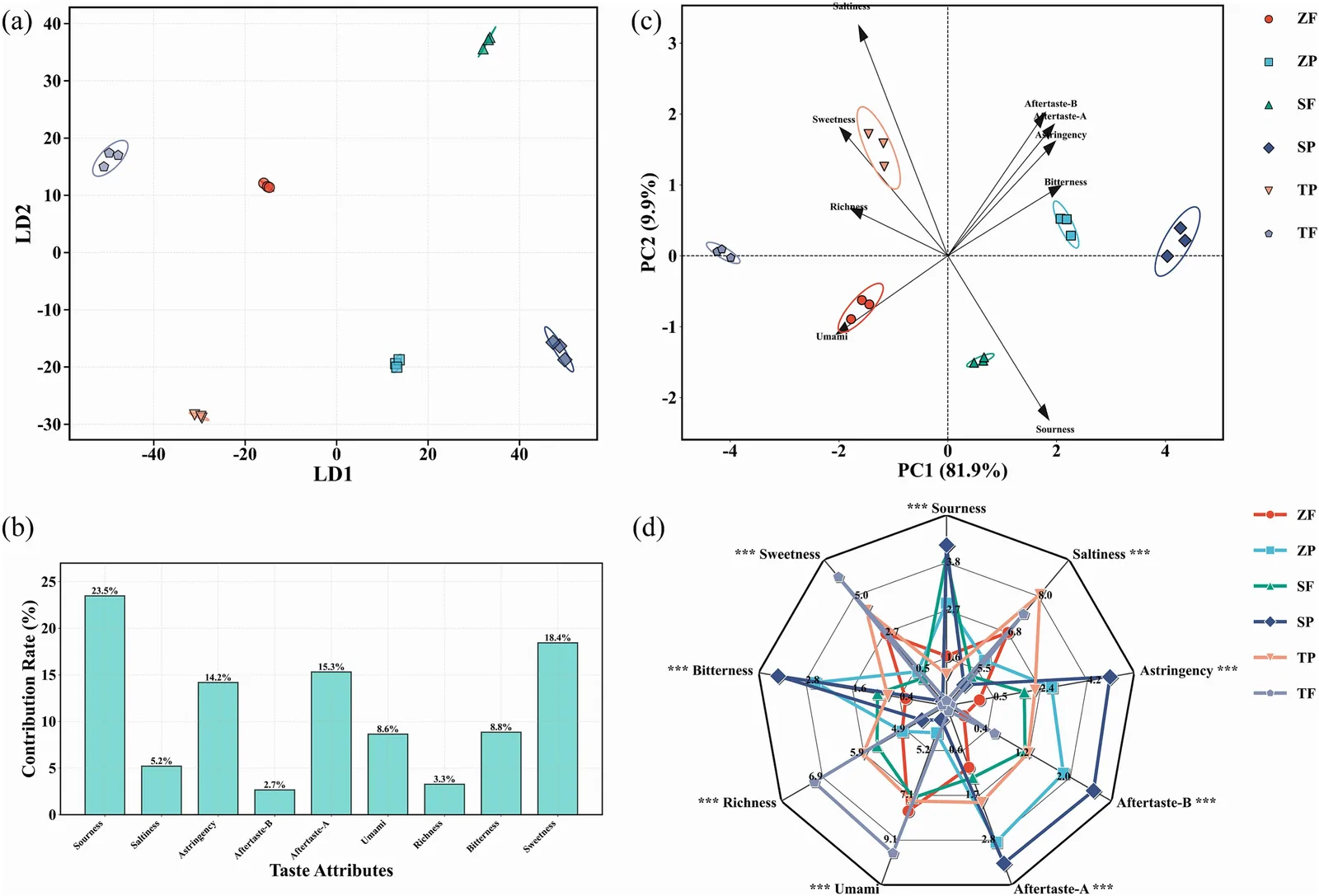
Electronic tongue (INSENT SA402B) taste profiling of peel and flesh tissues of three wampee (*Clausena lansium* (Lour.) Skeels) cultivars. (a) Linear discriminant analysis (LDA) of the nine-sensor taste response matrix for the six sample groups (SP, SF, TP, TF, ZP, ZF); (b) relative contribution (%) of each linear discriminant to the total discrimination; (c) principal component analysis (PCA) score plot of the *Z*-score-standardized taste responses (PC1 = 81.85%, PC2 = 9.91%); (d) radar plot of the mean taste intensity recorded for each of the nine taste sensors (AAE, CT0, CA0, GL1, C00, AE1, BT0, HA0, SW0) in each sample group. Sample abbreviations as in Fig. 1.

The complete separation achieved by LDA, together with the high variance explained by PCA, confirms that the six wampee sample groups possess distinct taste fingerprints that can be reliably discriminated by electronic tongue profiling. The dominance of sourness, sweetness, and aftertaste-astringency as discriminatory attributes aligns with the taste-active metabolite composition of wampee, where organic acids and soluble sugars are primary determinants of cultivar-specific sensory quality (Yin et al., 2022). The observed tissue-dependent sensory divergence—flesh exhibiting elevated sourness and sweetness while peel is enriched in astringency and bitterness—is consistent with recent metabolomics evidence that specialized metabolites accumulate preferentially in wampee peel, whereas sugars and organic acids partition predominantly into the flesh (Zhang et al., 2024). This tissue-specific partitioning has been similarly documented in other fleshy fruits, where peel and flesh serve distinct physiological functions reflected in their divergent phytochemical profiles (Xin et al., 2018). The strong loading of bitterness, umami, and sweetness on PC1 indicates that these attributes capture the primary axis of taste variation among wampee cultivars, mirroring phytochemical differences between sweet and sour varieties linked to distinct metabolite profiles (Chang et al., 2022). These results underscore the utility of electronic tongue systems coupled with multivariate chemometrics as rapid, objective tools for fruit quality assessment (Baietto & Wilson, 2015).

### 3.3 Aromatic properties of volatile components in different wampee varieties

Terpenoids constituted the dominant volatile class identified in wampee peel and pulp, accounting for 83 of the 288 detected compounds (28.8%), indicating that the terpenoid biosynthesis pathway is the primary metabolic route governing aroma formation in this species (Table S3). Esters (*n* = 45) and organoheterocyclic compounds (*n* = 31) represented the second and third most abundant classes, respectively, suggesting active fatty acid derivative metabolism and heterocyclic secondary metabolite synthesis. Hydrocarbons, alcohols, aldehydes, and ketones—key contributors to fruity and floral aroma notes—collectively comprised 87 compounds (30.2%), highlighting the chemical basis of wampee's distinctive sweet-tart flavor profile. The remaining classes, including phenolic derivatives and organosulfur compounds, were present at low abundances, reflecting tissue-specific metabolic specialization.

The volatile metabolome of three wampee cultivars comprised 15 chemical classes spanning terpenoids, esters, alcohols, hydrocarbons, and organoheterocyclic compounds, among which terpenoids constituted the dominant class at 28.82% of total volatiles across all six peel–flesh sample pairs (Fig. 3a). This terpenoid predominance aligns closely with the established volatile chemistry of Rutaceae family fruits, where mono- and sesquiterpenes synthesized via the MEP and MVA pathways typically account for the largest fraction of the essential oil profile (He et al., 2019; Singh et al., 2021). When resolved by tissue type and cultivar, terpenoid relative content exceeded 70% in five of the six samples (Fig. 3c) (Table S4), with only SF showing a modestly more balanced composition that included appreciable contributions from organoheterocyclic and ester fractions—a pattern consistent with the tissue-specific partitioning behavior documented in other fleshy fruits, where peel tissues accumulate higher concentrations of terpene-derived aroma compounds while flesh is comparatively enriched in alcohols and esters derived from primary metabolism (Arango et al., 2025; Dabbou et al., 2016). The sensory annotation of these volatiles revealed a broad aromatic palette spanning sweet (6.86%), fruity (3.92%), green (3.92%), citrus (2.94%), woody (2.78%), and balsamic (2.12%) descriptors (Fig. 3b), collectively reflecting a characteristic Rutaceae-type flavor architecture dominated by terpenic freshness and ester-driven fruitiness. Notably, the radar-profile comparison across the six samples exposed pronounced inter-cultivar divergence: SP and TP exhibited strong spicy and woody signatures, whereas TF and ZF were distinguished by elevated sweet and fruity attributes, and ZP showed a unique balsamic–citrus character (Fig. 3d). These cultivar-specific sensory fingerprints echo the chemotypic diversity reported across fruit species, where minor shifts in the relative abundance of key odor-active terpenes and esters can produce perceptibly distinct aromatic outcomes despite an otherwise conserved volatile class distribution (Sater et al., 2020).

**Fig. 3:**
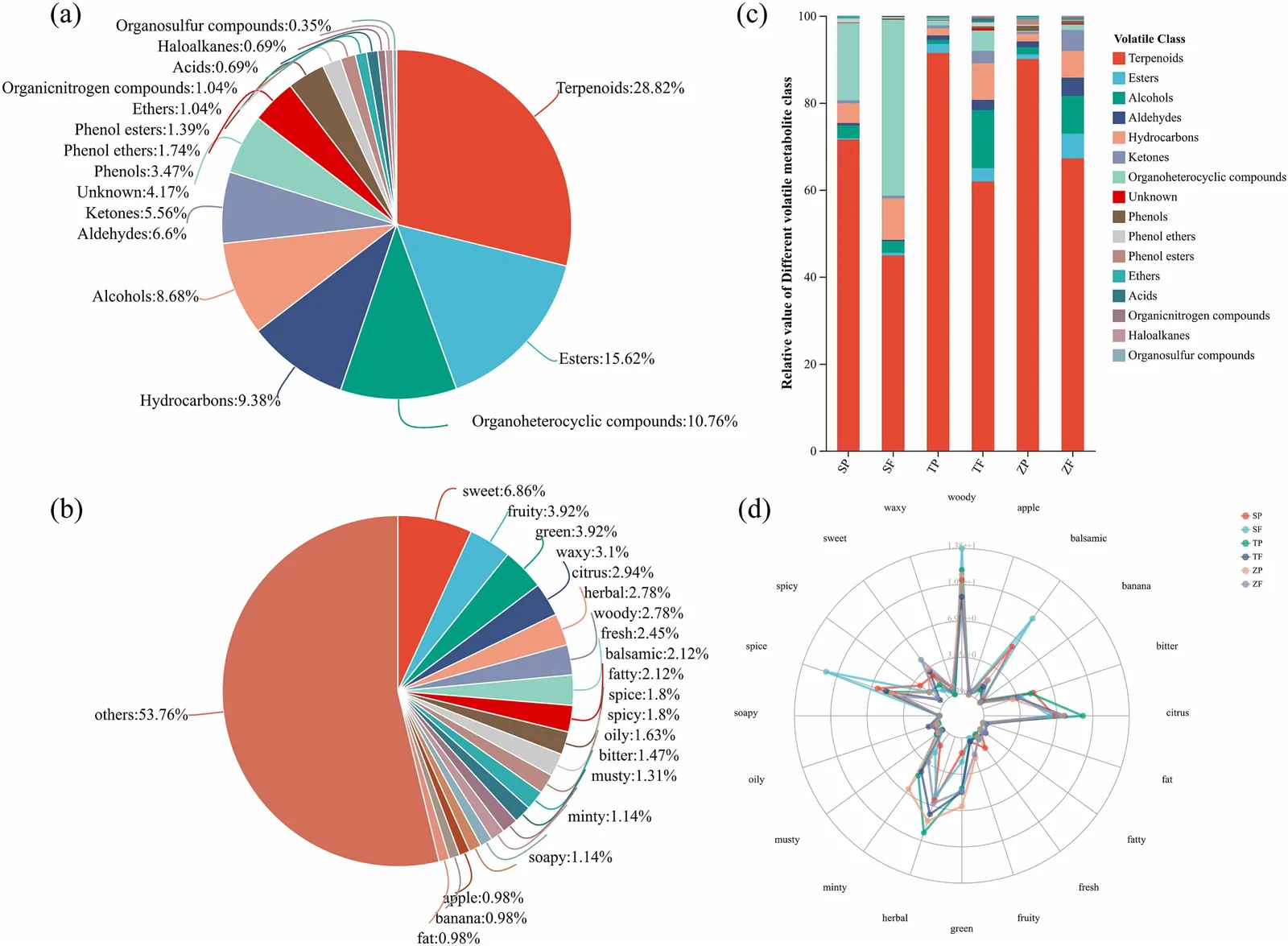
Composition and sensory annotation of the volatile metabolome in peel and flesh tissues of three wampee (*Clausena lansium* (Lour.) Skeels) cultivars. (a) Number and relative proportion (%) of the 288 identified volatile compounds classified into 15 chemical classes; (b) distribution of the volatile compounds across sensory flavor descriptors; (c) relative content (%) of each chemical class in the six sample groups (SP, SF, TP, TF, ZP, ZF); (d) radar plot of the relative content (%) of volatiles grouped by sensory flavor descriptor in each sample group. Sample abbreviations as in Fig. 1.

### 3.4 Aromatic characteristics of wampee

The OAV—the ratio of concentration to odor threshold—is a widely used metric for estimating a compound's potential contribution to food aroma. Compounds with OAV ≥ 1 are conventionally regarded as key odorants, and 57 such compounds were identified in this study. It should be noted that, because quantification was semi-quantitative (internal-standard-normalized peak areas with response factors assumed to be unity), the OAV values reported below are estimated relative values indicating relative—rather than absolute—odor potency, and cross-compound comparisons of OAV magnitude are constrained by this semi-quantitative basis. Their OAV profiles across the six samples (SP, SF, TP, TF, ZP, ZF) revealed markedly distinct patterns that reflect divergent volatile compositions (Table 3 and Table S5). The SP sample is overwhelmingly dominated by myristicin (safrole derivative; OAV = 2.86 × 10^6^), which alone accounts for over 89% of the total OAV in that sample and confers an intensely balsamic–woody–spicy character. Estragole (OAV = 2.56 × 10^5^ in SP) and damascenone (OAV = 2.16 × 10^5^ in SP) rank second and third, respectively, contributing sassafras-anise and tobacco-rose-honey notes that further define the SP aroma space. By contrast, the *Z*-series samples exhibit a markedly different hierarchy: estragole surges to its maximum OAV of 5.11 × 10^5^ in ZP, while 4-ethyl-2-methoxyphenol—a potent phenolic-smoke compound with the lowest odor threshold in the entire panel (0.01 μg L^−1^)—reaches OAV values of 3.20 × 10^4^ (ZP) and 4.94 × 10^4^ (ZF), strongly suggesting thermal processing or Maillard-derived smoke character in these matrices. Damascenone maintains consistently high OAVs across all six samples (range: 1.25 × 10^5^–4.55 × 10^5^), identifying it as a ubiquitous key odorant whose rose-tobacco signature likely forms a common aromatic backbone irrespective of sample origin. Green notes, represented by cis-3-hexenal (odor threshold = 0.09 ng L^−1^), emerge selectively in the ZF sample (OAV = 1.39 × 10^4^), implying preservation of fresh leafy character that is attenuated elsewhere. Although the ACI conveniently ranks odorants by their proportional share of the total OAV, the dominance of a single compound such as myristicin can render the remaining key odorants numerically negligible even when they make substantial perceptual contributions (Table S6). Importantly, OAV additivity does not predict perceptual additivity: in complex food matrices, mixture interactions such as perceptual synergy, suppression, and masking commonly occur.

**Table 3:** odor activity values (OAV) of the 57 odor-active volatile compounds quantified in peel and flesh of three wampee (*Clausena lansium* (Lour.) Skeels) cultivars.

No.	Volatile compound	CAS No.	SP	SF	TP	TF	ZP	ZF
1	Myristicin (4-Methoxy-6-(2-Propenyl)-1,3-Benzodioxole)	607-91-0	2863842.10	451410.77	75772.51	29882.37	28878.03	3772.89
2	Estragole	140-67-0	256329.33	1596.60	139145.90	72608.33	511733.36	2428.73
3	Damascenone	23726-93-4	215896.47	43049.69	288735.73	125516.36	455280.85	164448.68
4	(+/-)-Linalool	78-70-6	47567.59	2258.04	19710.09	9284.61	110531.48	16431.04
5	Myrcene	123-35-3	23783.14	1250.50	2740.11	157.41	1472.55	168.39
6	1,3-P-Menthadien-7-Al	1197-15-5	5058.70	3890.84	11224.26	4243.24	30246.07	2584.74
7	Nonanal	124-19-6	4570.53	1134.56	1057.81	2483.17	2831.06	3621.60
8	3,4-Dimethylphenol	95-65-8	2393.52	61.10	497.45	243.16	1493.34	89.02
9	Cis-3-Hexenal	6789-80-6	1251.99	282.93	397.15	224.30	3735.39	13906.73
10	Eugenol	97-53-0	963.22	89.46	291.11	50.74	1599.78	486.54
11	Butyl Acetate	123-86-4	957.60	5.76	18421.33	34.89	513.69	562.97
12	(+/-)-4-Terpineol	562-74-3	939.19	1.27	1032.68	15.98	1343.75	22.15
13	Alpha-Terpinene	99-86-5	813.62	40.52	423.69	22.73	386.87	22.45
14	2-Nonenal	18829-56-6	635.77	162.22	1288.06	19199.57	4075.52	3468.13
15	Carvacrol	499-75-2	591.91	10.74	14.80	10.24	560.08	13.53
16	M-Xylene	108-38-3	585.02	0.90	53.74	3.66	1187.54	2.03
17	Ethyl Salicylate	118-61-6	570.60	11.50	13.23	1.58	75.83	0.22
18	3-Octanone	106-68-3	511.80	13.54	12.53	10.51	112.72	58.71
19	Alpha-Phellandrene	99-83-2	488.93	64.84	1154.18	89.73	2174.68	61.16
20	P-Cresol	106-44-5	392.43	55.94	57.74	82.23	2470.49	101.93
21	4-Ethyl-2-Methoxyphenol	2785-89-9	317.39	65.24	103.46	197.16	31999.64	49436.92
22	Rose Oxide	3033-23-6	285.08	20.86	60.06	79.71	905.37	1575.07
23	Cis-Nerolidol	142-50-7	229.07	4.43	44.36	16.98	276.73	10.20
24	Octanal	124-13-0	225.84	0.37	0.81	0.67	6.67	0.93
25	Indole	120-72-9	120.83	29.81	125.06	121.28	211.50	73.10
26	2-Methylnaphthalene	91-57-6	95.08	8.57	100.35	55.84	110.81	18.43
27	2-Nonanone	821-55-6	94.33	26.68	8.09	59.28	19.57	23.80
28	Phenylethyl Alcohol	60-12-8	91.39	10.42	1695.11	835.51	5844.37	1325.52
29	Alpha-Terpineol	98-55-5	87.77	12.72	90.59	10.20	310.68	8.13
30	Decanal	112-31-2	81.86	84.05	135.78	346.67	164.50	307.52
31	(+/-)-Beta-Citronellol	106-22-9	80.52	3.40	114.49	131.64	13716.77	16.11
32	2,4-Decadienal	25152-84-5	75.03	38.25	119.35	120.09	228.09	230.20
33	(4Z)-4,11,11-Trimethyl-8-Methylidenebicyclo [7.2.0] Undec-4-Ene	87-44-5	74.55	0.01	83.34	0.25	153.16	0.86
34	2,4-Hexadienal	142-83-6	66.20	155.18	35.02	352.01	546.14	165.05
35	2,4-Nonadienal	5910-87-2	41.37	4.24	113.59	59.25	171.03	187.58
36	Ethanol	64-17-5	37.94	21.88	32.83	171.22	273.52	121.50
37	Geraniol	106-24-1	17.12	0.70	9.26	1.51	18.21	1.72
38	1-Butanol	71-36-3	15.87	0.20	8.61	3.16	172.39	253.49
39	Cumene	98-82-8	15.03	0.15	3.16	0.74	9.83	0.31
40	Ethylbenzene	100-41-4	12.03	0.56	0.53	0.73	3.55	1.81
41	Gamma-Terpinene	99-85-4	7.11	0.15	1.51	0.44	3.98	0.32
42	Galaxolide	1222-05-5	6.72	2.97	37.20	7.36	1080.97	7.84
43	Ethyl Octanoate	106-32-1	6.43	0.91	12.37	25.16	107.30	31.58
44	Isobutyl Butyrate	539-90-2	5.84	6.12	2.94	1.87	71.17	3.78
45	Styrene	100-42-5	4.48	<0.01	0.01	0.10	0.50	0.22
46	O-Xylene	95-47-6	4.37	0.13	2.39	0.25	3.94	0.28
47	Amyl Salicylate	2050-08-0	3.56	4.68	0.84	2.23	13.15	3.52
48	Ethyl Acetate	141-78-6	3.47	2.76	4.61	7.70	108.05	33.55
49	2-Octenal	2548-87-0	2.29	0.46	5.37	17.85	8.91	13.45
50	Cyclohexanol	108-93-0	1.10	0.09	1.33	1.85	6.27	1.55
51	P-Aminotoluene	106-49-0	1.04	0.53	0.28	33.60	4.70	12.55
52	Dibutyl Phthalate	84-74-2	0.74	0.64	1.40	2.13	3.71	2.04
53	Hexyl Acetate	142-92-7	0.72	0.07	36.52	11.30	120.26	62.07
54	3-(Methylthio)Propionaldehyde	3268-49-3	0.44	0.03	0.49	11.60	4.10	3.61
55	1-Pentanol	71-41-0	0.38	0.28	0.11	1.39	8.19	2.80
56	1,7,7-Trimethylbicyclo[2.2.1]Heptan-2-Ol	507-70-0	0.32	0.51	6.39	9.96	25.10	15.10
57	Dibutyl Ether	142-96-1	0.19	0.02	0.72	0.51	2.61	1.36

Note: The values reported in this table are odor activity values (OAV), i.e. the dimensionless ratio of the measured concentration of each compound to its odor detection threshold in air (thresholds expressed in μg L^−1^). An OAV > 1 indicates that the compound makes a significant contribution to the overall odor profile. Values are dimensionless ratios of the semi-quantitatively determined concentration of each compound to its odor detection threshold in air. Sample abbreviations as in Table 1.

### 3.5 Exploratory analysis of volatile components

Multivariate statistical analysis of 28 volatile metabolites across the six wampee sample groups revealed pronounced inter-group divergence that was consistently captured by three complementary approaches (Fig. 4). The PLS-DA scores plot demonstrated that Component 1 (51.6%) and Component 2 (20.5%) collectively explained 72.1% of the total variance, with the Shanhuangpi peel (SP) samples occupying a discrete position in the upper-right quadrant that was fully resolved from the aggregated cluster comprising SF, TP, TF, ZP, and ZF (Fig. 4a). This segregation indicates that SP possesses a fundamentally distinct volatile metabolic architecture relative to all other cultivar–tissue combinations—a finding independently corroborated by principal component analysis, where PC1 (51.70%) and PC2 (20.59%) reproduced an equivalent separation pattern with SP isolated at the positive extreme of PC1 (Fig. 4b). The sample-to-sample correlation heatmap further validated this dichotomy: biological replicates within each group exhibited strong positive correlations (*r* > 0.90, **P < 0.001), confirming analytical reproducibility, whereas between-group correlation coefficients disclosed a block-diagonal structure in which SP replicates were negatively or weakly correlated with most non-SP groups, particularly SF (Fig. 4c). Notably, TP, TF, ZP, and ZF formed an intermediate correlation continuum rather than discrete clusters, suggesting that these four groups share a common volatile backbone despite cultivar-level quantitative differences. The exceptional separation of SP likely reflects its enrichment in phenylpropene-derived volatiles—notably safrole and estragole—which have been previously documented as signature constituents of *Clausena lansium* peel essential oil (He et al., 2019). Collectively, these multivariate results establish that volatile metabolic divergence among wampee cultivars is driven primarily by the SP outlier profile, while the remaining five sample groups occupy a more compact metabolomic space whose internal structure is shaped by subtle tissue- and cultivar-dependent modulations rather than wholesale compositional reorganization.

**Fig. 4:**
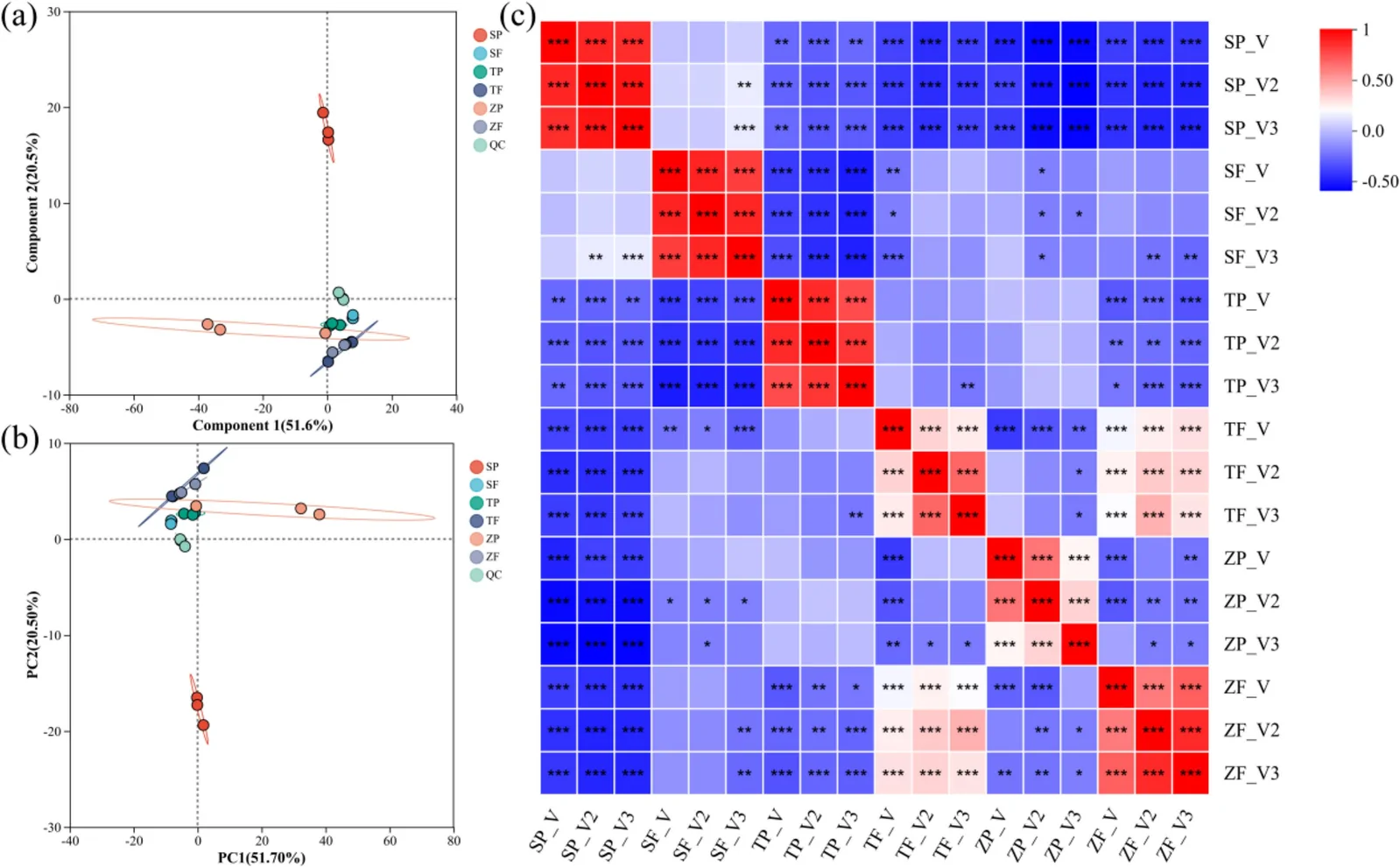
Inter-cultivar multivariate analysis of the volatile metabolome of the six wampee (*Clausena lansium* (Lour.) Skeels) sample groups. (a) Partial least-squares discriminant analysis (PLS-DA) score plot of the inter-cultivar model (Component 1 = 51.6%, Component 2 = 20.5%); (b) principal component analysis (PCA) score plot of the same metabolomic dataset (PC1 = 51.70%, PC2 = 20.59%); (c) sample-to-sample Pearson correlation heatmap showing the agreement between biological replicates within each group and the divergence between groups. Sample abbreviations as in Fig. 1.

### 3.6 Screening of metabolites with inter-variety differences

Volatile profiling across wampee (*Clausena lansium*) cultivars revealed pronounced divergence in both the composition and relative abundance of aroma-active compounds. The VIP bubble plot and heatmap of the top 50 volatiles (VIP > 1.1) showed that sesquiterpenes—notably β-bisabolene, caryophyllene, and β-sesquiphellandrene—formed the most discriminating class, consistent with the sesquiterpene-dominated profile reported for wampee pericarp and leaf (Fig. 4a). Cultivar-specific enrichment of monoterpenes (sabinene, α-pinene, myrcene) suggests that the relative flux through the plastidial methylerythritol phosphate (MEP) versus cytosolic mevalonate (MVA) pathway differs among cultivars. HMDB classification indicated that terpenoids and lipid-like molecules together dominated the discriminant volatiles, with organoheterocyclic compounds forming a secondary cluster (Fig. 4b); this terpenoid dominance parallels other fruit-species surveys where such investment is linked to ecological interactions and oxidative-stress mitigation. In wampee, the cultivar-driven divergence in peel-versus-pulp phenolic accumulation—mediated by phenylpropanoid and flavonoid biosynthesis—further corroborates the tissue-specific metabolic specialization seen in the volatile dataset. KEGG enrichment of the volatile set identified terpenoid backbone biosynthesis, monoterpenoid biosynthesis, and sesquiterpenoid/triterpenoid biosynthesis as the leading pathways, together with phenylpropanoid biosynthesis and α-linolenic acid metabolism (Fig. 5c; Mostafa et al., 2022). Comparable multi-omics dissection of terpenoid biosynthesis in *Zanthoxylum armatum* fruits has linked terpenoid pathway gene expression directly to the accumulated terpenoid pool (Yang et al., 2026).

**Fig. 5:**
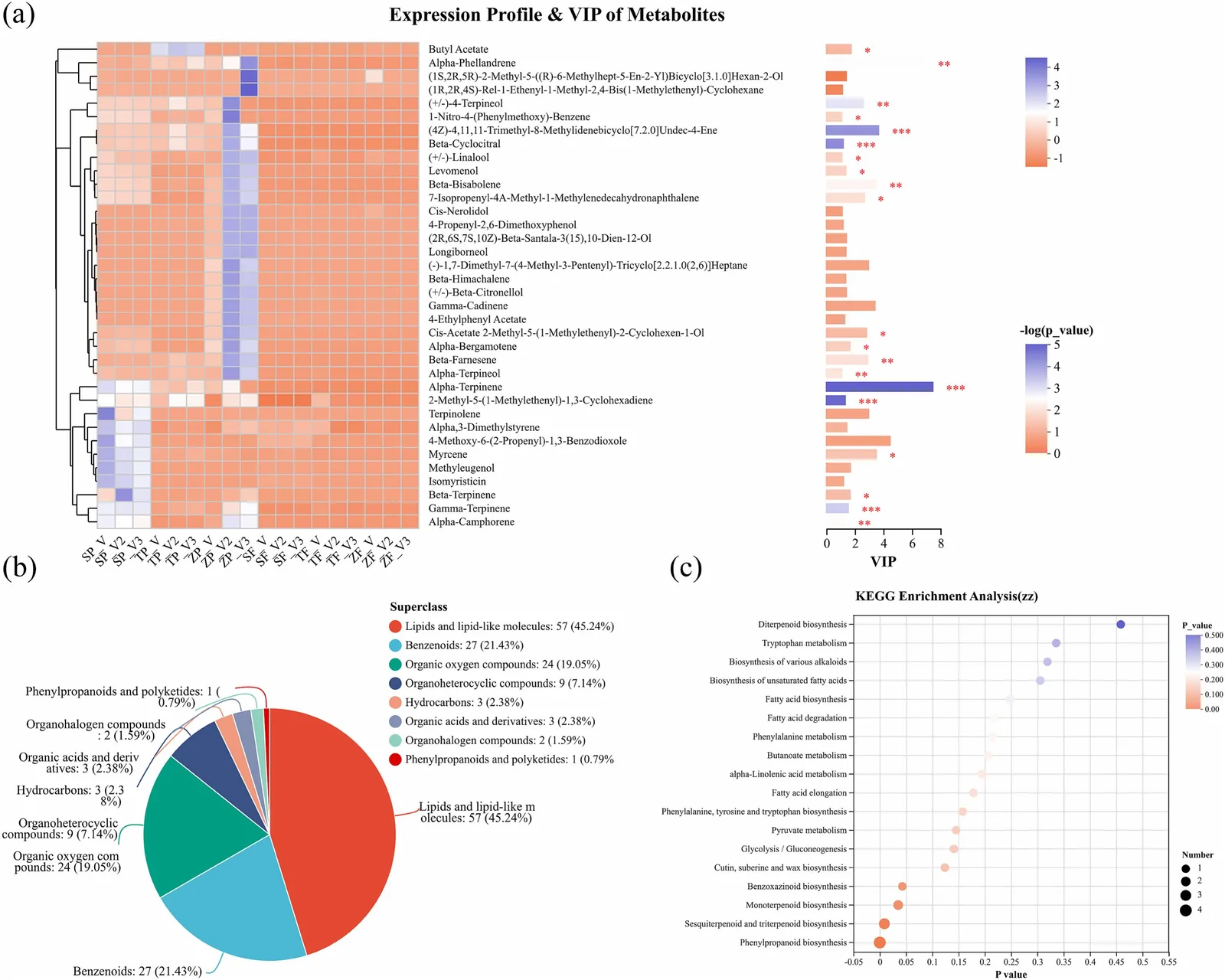
Differential volatile metabolites and their functional annotation in wampee (*Clausena lansium* (Lour.) Skeels) cultivars. (a) VIP bubble plot and heat map of the relative abundance of the top 50 discriminant volatile metabolites (VIP > 1.1) across the six sample groups. In the left-hand panel, metabolites are arranged on the y-axis and sorted in descending order of VIP value along the x-axis; in the right-hand panel, each column represents one sample group (labelled beneath the plot) and each row one metabolite, with colour intensity proportional to the relative abundance of that metabolite in the corresponding sample group, as indicated by the colour scale bar. (b) HMDB hierarchical classification of the discriminant volatile metabolites. (c) KEGG pathway enrichment analysis of the discriminant volatile metabolites.

These assignments are mechanistically coherent. The peel-enriched accumulation of monoterpenes and phenylpropenes (myristicin, estragole) is consistent with differential engagement of the plastidial MEP pathway (supplying IPP/DMAPP for monoterpenes) and the cytosolic MVA pathway (feeding sesquiterpenes), whose prenyl diphosphate precursors are funnelled into skeletons by terpene synthases (TPS) and diversified into specific odorants—linalool and myrcene via cytochrome P450 monooxygenases and glycosyltransferases, and apocarotenoids such as damascenone via carotenoid-cleavage dioxygenases (CCD) (Mostafa et al., 2022). Phenylpropenes branch from the phenylpropanoid pathway downstream of phenylalanine ammonia-lyase (PAL), whereas green-leaf volatiles such as cis-3-hexenal arise from the α-linolenic acid → lipoxygenase (LOX) cascade. Known regulatory nodes—*DXS*, *DXR*, *HMGR*, *TPS*, *PAL*, *LOX*—are plausible drivers of the observed flux, and candidate transcription factors of the *MYB*, *bHLH*, and *AP2*/*ERF* families may govern the cultivar-specific distribution of carbon between these branches (Mostafa et al., 2022). That microbial activity can reshuffle a plant volatile metabolome in a directed, branch-specific manner is independently illustrated by solid-state fermentation of dark teas with different Bacillus species (Yang, Ding, et al., 2025), by strain-resolved volatile fingerprints during *Eurotium cristatum* fermentation (Xiao et al., 2022), including co-culture with water kefir (Gao et al., 2025), and by parallel multi-platform decoding in Moso bamboo shoots across growth stages (Zhang, Peng, et al., 2025). Comparable genotype- and process-dependent volatile divergence has been documented across other fermented food matrices, ranging from cocoa (Murti et al., 2025) and aromatic coconut cider (Songprasert et al., 2025) to aquatic and meat products (Xu et al., 2025; Yang, Lu, et al., 2025).

The consistent peel bias of terpenoids and phenylpropenes is biologically plausible because the exocarp serves as a protective outer tissue: in many Rutaceae fruits, the oil-gland-rich flavedo stores volatile defense compounds that deter herbivores and pathogens and afford UV protection (Rodríguez et al., 2015), whereas the flesh is preferentially partitioned toward sugars, organic acids, and C₆ aldehydes/alcohols from primary metabolism (Lin et al., 2023; Mostafa et al., 2022). Direct evidence for a defensive role of these volatiles in wampee, however, awaits dedicated ecological or gland-localized metabolomic assays.

We stress that KEGG enrichment performed solely on detected volatile metabolites provides only weak, indirect evidence for actual pathway activity or metabolic flux. Because many intermediates are non-volatile and were not measured, enrichment reflects the presence of end-products rather than enzyme abundance, catalytic rate, or gene expression; statements such as “MEP engagement in peel” or “MVA activity in pulp/seed” should therefore be read as compatible with, not demonstrative of, the underlying flux (Mostafa et al., 2022). The analysis is further confined to the top 50 VIP-selected volatiles. Full-scan untargeted metabolomics with authentic standards, coupled with GC–O and sensory-panel validation, plus integrative transcriptomic–metabolomic or enzyme-activity studies across multiple cultivars and developmental stages, will be required to confirm the regulatory networks—particularly *MYB/bHLH/AP2-ERF*—that orchestrate cultivar- and tissue-specific volatile biosynthesis in wampee.

### 3.7 Aroma analysis of characteristics across different varieties

Significant inter-cultivar variations were observed in all tested physicochemical parameters. Bingtangxin had the highest pH and sugar-acid ratio with the lowest titratable acid content. Heijingang showed the highest soluble solids content, with a soluble sugar level comparable to Bingtangxin. Shanhuangpi exhibited the highest titratable acid and the lowest sugar-acid ratio (Table S7). The integrated sensory flavor profiling of wampee (*Clausena lansium*) cultivars revealed cultivar-specific architecture of aroma-active compounds whose OAV directly translated into differential sensory perception (Fig. 6a–d). The OAV line chart (Fig. 6a) demonstrated that compounds with OAV > 1—those exceeding the odor detection threshold and thus capable of contributing directly to the overall aroma profile—were dominated by terpenoids (notably linalool, α-terpineol, and β-caryophyllene) and C₆ aldehydes (hexanal, (E)-2-hexenal), whose elevated OAV magnitudes distinguished cultivars with pronounced fruity, floral, and green notes. In contrast, compounds with OAV < 1, although measurable by GC–MS, exerted no appreciable sensory impact, underscoring the importance of threshold-weighted metrics over raw abundance when identifying cultivar-defining aroma signatures. The aroma characteristic level (ACL) analysis (Fig. S2) further resolved the dominant sensory dimensions—green, fruity, floral, sweet, and woody—across cultivars, with the green (aldehyde-driven) and fruity (ester-driven) dimensions exhibiting the greatest inter-cultivar variation, a pattern broadly consistent with multi-platform aroma profiling in strawberry cultivars (Zhang, Yan, et al., 2025). The sensory flavor compound count statistics (Fig. 6b) revealed that cultivars with a broader repertoire of OAV > 1 compounds, particularly those enriched in ester and monoterpene classes, received higher ACL scores across multiple sensory dimensions, suggesting that aroma complexity rather than the dominance of a single volatile class drives the overall sensory appeal. The Sankey diagram (Fig. 6c) visualized the flow of volatile metabolites from chemical superclass to odor descriptor to cultivar assignment, revealing that terpenoids mapped preferentially to floral and woody notes, whereas aldehydes and esters mapped to green and fruity notes, respectively, with cultivar-specific branching patterns that reflect differential biosynthetic pathway engagement. This flow-based visualization highlights that the same odor descriptor (e.g., “fruity”) can be contributed by chemically distinct volatile pools—esters in one cultivar versus lactones in another—emphasizing convergent sensory outcomes from divergent metabolic routes. The combined ACL and OAV framework employed here extends beyond conventional volatile profiling by weighting compound abundance against sensory thresholds, a strategy that has proven effective for deconvoluting aroma complexity in peach fruit (Zhu et al., 2026). Notably, the cultivar possessing the highest total volatile abundance did not necessarily exhibit the strongest sensory impact, as several high-abundance volatiles fell below their odor thresholds, a phenomenon similarly reported in *Actinidia arguta* cultivar surveys (Xu et al., 2024). This decoupling of chemical abundance from sensory contribution reinforces the necessity of OAV-driven compound prioritization in aroma characterization workflows.

**Fig. 6:**
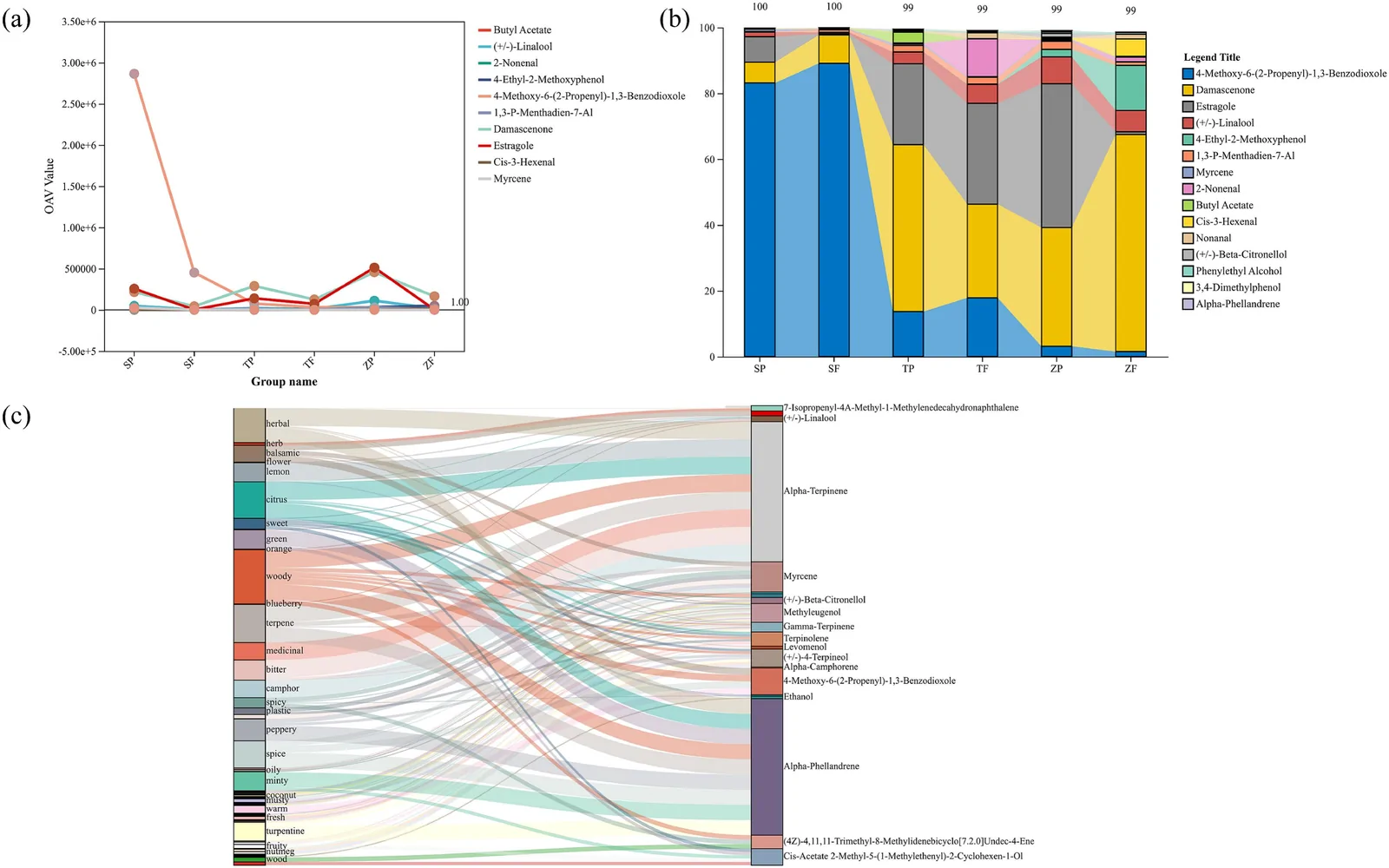
Sensory flavor characteristics of three wampee (*Clausena lansium* (Lour.) Skeels) cultivars. (a) Distribution of the odor activity values (OAV) of the 57 quantitated volatile compounds across the six sample groups (SP, SF, TP, TF, ZP, ZF); (b) number of sensory flavor-active volatile compounds (OAV > 1) assigned to each sensory descriptor; (c) Sankey diagram linking chemical superclass, sensory flavor descriptor and sample group for the volatile compounds with OAV > 1. Sample abbreviations as in Fig. 1. Note: In panel (a), different colours on the abscissa denote distinct sample groups, the ordinate corresponds to OAV, and each data point represents one volatile compound. An OAV < 1 indicates that the compound makes no practical contribution to the overall odor, whereas an OAV > 1 indicates that it may exert a direct influence on the overall odor profile. Sample abbreviations as in Fig. 1.

To further link the electronic-sensing responses with volatile composition, we performed Spearman correlation analyses between the sensor/taste outputs and the 57 GC–MS-identified volatile compounds (Fig. S3). W1W, nominally a sulfur sensor, correlated most strongly with phenolics such as 3,4-dimethylphenol (*p* < 0.01) and ethyl salicylate (*p* < 0.01) rather than with sulfur-containing volatiles. Hierarchical clustering grouped terpenes and phenolics into a coherent block that correlated positively with W5S, W1S, W2W, and W3S. These patterns indicate that the MOS sensors register composite volatile profiles rather than discrete chemical classes, which accounts for the strong W1W/W1S discrimination despite the low abundance of sulfur/methyl compounds in the GC–MS dataset. In Fig. S4, astringency and aftertaste attributes clustered with terpenic and phenolic volatiles, showing strong positive correlations with carvacrol (*p* < 0.01), myrcene, and α-terpinene. Ethyl salicylate correlated positively with aftertaste-bitter and astringency but negatively with umami (*p* < 0.05). Richness was inversely associated with 3-octanone (*p* < 0.001) and eugenol. The clustering indicates that taste perception is partly shaped by specific volatile classes, supporting an integrated multi-platform flavor-omics approach.

### 3.8 Screening of differential metabolites between fruit peel and pulp

To identify tissue-specific volatile metabolites that distinguish wampee peel from flesh, partial least squares-discriminant analysis (PLS-DA) was applied to the complete volatile profile dataset comprising all six sample pairs. The resulting scores plot demonstrated clear separation between peel (P) and flesh (F) groups along the first two components (Component 1: 53.20%; Component 2: 30.70%) with no overlap between 95% confidence ellipses (Fig. 7a). The robustness of this discrimination was confirmed by permutation testing (*n* = 200), which yielded cumulative R^2^Y = 0.833 and Q^2^ = 0.647—values that satisfy established criteria for a valid PLS-DA model wherein R^2^Y exceeds Q^2^ and both are well above zero, indicating that the observed separation is not due to chance overfitting (Fig. 7b). Principal component analysis independently corroborated this tissue dichotomy, with PC1 (51.80%) and PC2 (21.01%) collectively explaining 72.81% of total variance and cleanly segregating peel from flesh samples along the PC1 axis (Fig. 7c). The volcano plot of differential volatiles identified 96 compounds significantly up-regulated in peel relative to flesh (log₂FC > 0, *p* < 0.05; red) and 6 compounds down-regulated (log₂FC < 0, *p* < 0.05; blue) (Fig. 7d). Variable importance in projection (VIP) analysis further highlighted that monoterpene hydrocarbons—including α-terpinene, geraniol, β-cyclocitral, and bicyclic sesquiterpene derivatives such as 4,7,7-trimethylbicyclo[4.1.0]hept-3-ene—ranked among the top contributors to peel–flesh discrimination, underscoring the central role of terpenoid metabolism in defining the aromatic identity of each tissue layer.

**Fig. 7:**
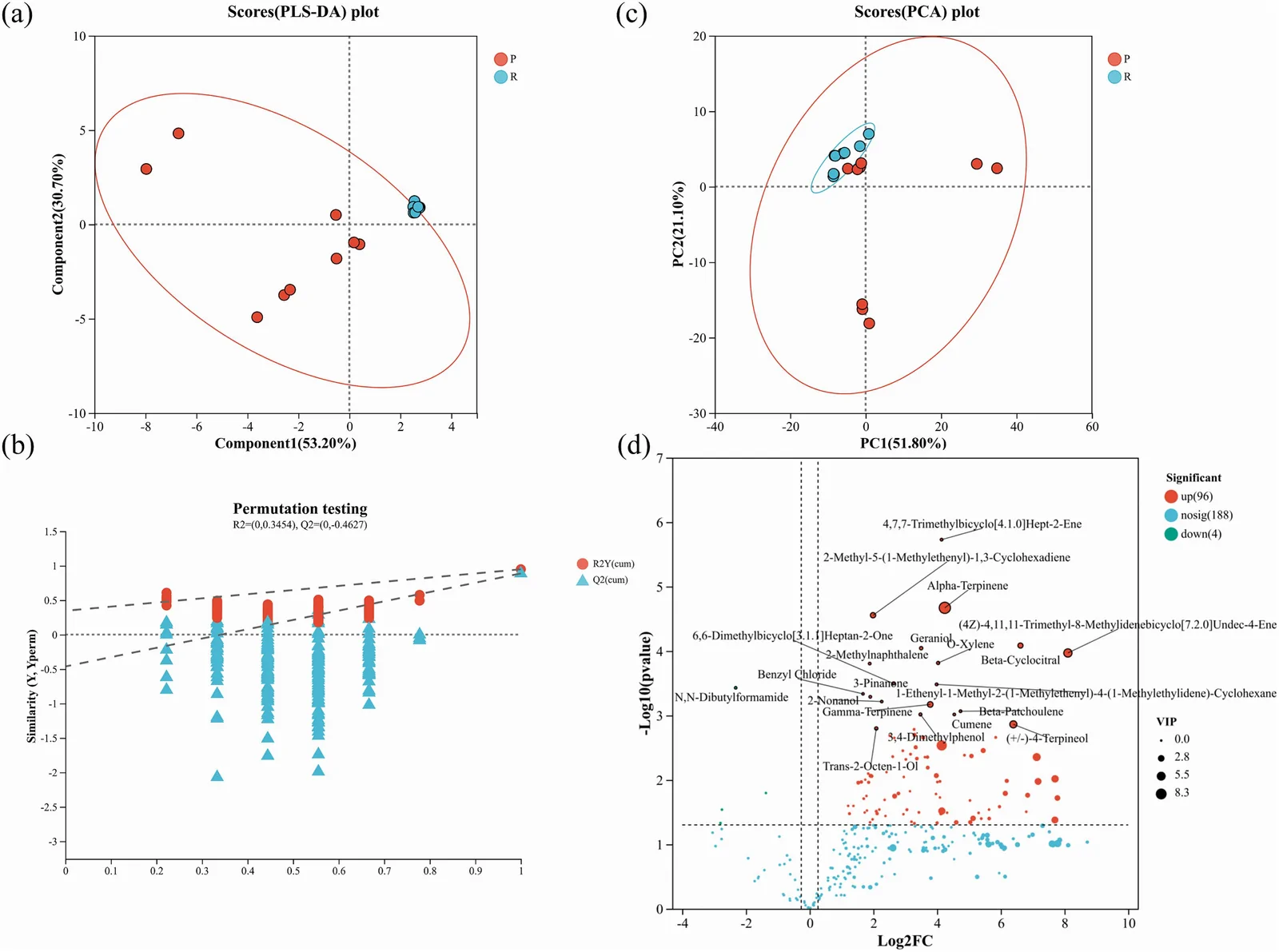
Multivariate statistical analysis of tissue-specific volatile metabolites in peel and flesh of three wampee (*Clausena lansium* (Lour.) Skeels) cultivars. (a) PLS-DA score plot of peel (P, red) and flesh (F, blue) samples; the 95% confidence ellipses show complete separation between the two tissue groups with no overlap (Component 1 = 53.20%, Component 2 = 30.70%); (b) permutation test of the PLS-DA model (*n* = 200; R^2^Y = 0.833, Q^2^ = 0.647); (c) PCA score plot of the same dataset (PC1 = 51.80%, PC2 = 21.01%); (d) volcano plot of the differentially abundant volatile metabolites between peel and flesh. Red dots represent metabolites significantly up-regulated in peel (*n* = 96; log₂FC > 0, *p* < 0.05), blue dots represent metabolites significantly down-regulated in peel (*n* = 6; log₂FC < 0, *p* < 0.05), and cyan dots represent non-significant metabolites (*n* = 186). Dot size reflects the variable importance in projection (VIP) score, with larger dots indicating higher VIP values. Sample abbreviations as in Fig. 1. (For interpretation of the references to colour in this figure legend, the reader is referred to the web version of this article.)

Integration of these multivariate results with odor activity value (OAV) data (Table 3) reveals a coherent picture of tissue-specific aroma architecture. Peel samples across all three cultivars were characterized by exceptionally high OAVs for safrole-derived phenylpropenes—notably myristicin (SP OAV = 2.86 × 10^6^) and estragole (ZP OAV = 5.12 × 10^5^)—together with the norisoprenoid damascenone (ZP OAV = 4.55 × 10^5^) and the acyclic monoterpenes linalool (ZP OAV = 1.11 × 10^5^) and myrcene (SP OAV = 2.38 × 10^4^). These peel-enriched odorants collectively confer a balsamic–woody–spice aroma signature consistent with the known accumulation patterns of terpenoids and phenylpropanoid-derived essential oil constituents in fruit exocarp tissues (Lafuente et al., 2021). By contrast, flesh tissues exhibited markedly elevated OAVs for green leaf volatiles—cis-3-hexenal (ZF OAV = 1.39 × 10^4^) and 2-nonenal (TF OAV = 1.92 × 10^4^)—as well as for rose-related monoterpenes including rose oxide (ZF OAV = 1.57 × 10^3^), β-citronellol (ZP OAV = 1.37 × 10^4^), and phenylethyl alcohol (TP OAV = 1.70 × 10^3^), which together produce a fresher, sweeter, and more floral sensory character. This peel-versus-flesh partitioning pattern closely mirrors findings in lemon, cherry, and other fleshy fruits where peel tissues preferentially accumulate terpenoid-rich essential oils while flesh is comparatively enriched in C₆ aldehydes, alcohols, and ester-type volatiles arising from primary metabolic pathways (Li et al., 2026; Liu et al., 2026). Notably, the phenolic compound 4-ethyl-2-methoxyphenol—an intensely smoky odorant with an ultralow threshold of 0.01 μg L^−1^—achieved its highest OAV values in the *Z*-series samples regardless of tissue (ZP: 3.2 × 10^4^; ZF: 4.9 × 10^4^), suggesting a cultivar-specific rather than tissue-specific origin for this Maillard-derived volatile. Together, these results demonstrate that the characteristic aroma of wampee arises from a relatively small subset of key odorants whose tissue-specific distribution is governed by distinct biosynthetic routes: terpenoid and phenylpropanoid pathways dominate in the peel, whereas fatty acid degradation and glycoside hydrolysis contribute disproportionately to the flesh volatile bouquet—a conclusion fully consistent with OAV-based aroma reconstruction studies that identify 8–15 key odorants as sufficient to explain >90% of perceived fruit aroma (Zhu et al., 2018; Qi et al., 2020; Tan et al., 2022).

### 3.9 Novelty and limitations

This is the first study to integrate *E*-nose, E-tongue, and HS-SPME-GC–MS flavor-omics to resolve peel-versus-flesh aroma in wampee, and to propose five cultivar-independent key odorants (myristicin, estragole, damascenone, linalool, myrcene) as candidate quality markers. Several limitations should be noted. (i) All aroma and taste conclusions rest on instrumental measurements; no human sensory panel (profiling or GC-O) was performed, so the perceptual reality of the inferred notes and the validity of OAV-based rankings remain unconfirmed. (ii) OAVs are semi-quantitative (internal-standard-normalized peak areas) and depend on odor thresholds that vary by orders of magnitude across reporting matrices; absolute odor potencies should therefore be interpreted cautiously. (iii) KEGG pathway inferences derive from volatile end-products alone and exclude non-volatile intermediates. (iv) The HS-SPME extraction temperature (80 °C) may induce thermal degradation or artefact formation for labile compounds. (v) Biological replication was limited to three trees per cultivar; given the high natural variation in fruit volatiles, subtle cultivar effects may be underpowered, and a formal power analysis was beyond the present scope. Addressing these gaps—through sensory validation, multi-omics integration across developmental stages, and expanded replication—is essential before the proposed markers are deployed in breeding or grading.

## 4 Conclusion

In summary, this study employed a multi-platform integrated flavor-omics strategy to systematically characterize the volatile profiles and taste attributes of three wampee (*Clausena lansium*) cultivars across peel and flesh tissues, providing new insights into the aroma and taste architecture. The main conclusions are as follows:

(i) The three wampee groups are robustly separable by biomimetic sensing, and 288 volatiles (28.82% terpenoids) were identified. Five OAV > 1 odorants—myristicin, estragole, damascenone, linalool, and myrcene—emerge as candidate chemical markers whose relative abundances define the balsamic-floral-spicy-woody-fruity wampee aroma and could support rapid aroma-based screening and quality control.
(ii) Myristicin, estragole, damascenone, linalool, and myrcene provide rapid, objective chemical markers for quality grading, cultivar authentication, and quality screening. Astringency/bitterness-enriched peel is directed toward extraction and value-added processing (essential oils, fortified products), whereas sweetness/sourness-rich flesh is reserved for fresh and dessert markets.
(iii) Future work should pursue transcriptomic–metabolomic integration across developmental stages to identify master regulators of terpenoid flux and trace aroma formation to its genetic origin, and apply GC–O coupled with recombination/omission studies to perceptually validate the five key odorants.

Collectively, these findings establish a scientific foundation for wampee quality assessment, cultivar authentication, and geographical origin tracing, with practical implications for flavor-oriented breeding, tissue-specific optimization of processing techniques, and objective commercial quality-grading criteria—offering actionable guidance for precise resource exploitation and value enhancement of this edible-medicinal tropical fruit tree.

## CRediT authorship contribution statement

**Qingshan Li:** Writing – review & editing, Writing – original draft, Funding acquisition, Formal analysis, Data curation. **Chunyan Zhang:** Writing – review & editing, Methodology, Formal analysis, Data curation. **Ruibing Xu:** Writing – original draft, Validation, Conceptualization. **Gengrui Zhu:** Data curation, Conceptualization. **Yi Chen:** Funding acquisition, Formal analysis. **Haidong Huang:** Software, Resources. **Gaoyang Zhang:** Writing – review & editing, Supervision, Project administration, Funding acquisition.

## Declaration of competing interest

The authors declare that they have no known competing financial interests or personal relationships that could have appeared to influence the work reported in this paper.

## Data Availability

Data will be made available on request.
