# Supplementary material for: Integrating E-nose, E-tongue, and HS-SPME-GC–MS analysis reveals the volatile profile and flavor characteristics of different Wampee (Clausena lansium (Lour.) Skeels)
Source: Food Chem X. 2026 Oct 28;40:104660. doi: 10.1016/j.fochx.2026.104660 (PMC13742506; doi:10.1016/j.fochx.2026.104660)
Supplement: Supplementary file 1 [file mmc1.docx]

**Figure S1** Total lon Chromatograms (TIC) of six wampee cultivars

**Figure S2** ACL result plot.

**Figure S3** eNose vs Volatiles correlation heatmap

* *p* < 0.05, ***p* < 0.01, and ****p* < 0.001

**Figure S4** eTongue vs Volatiles correlation heatmap

* *p* < 0.05, ***p* < 0.01, and ****p* < 0.001
